# Ferroptosis of CD163^+^ tissue-infiltrating macrophages and CD10^+^ PC^+^ epithelial cells in lupus nephritis

**DOI:** 10.3389/fimmu.2023.1171318

**Published:** 2023-07-31

**Authors:** Qi Cheng, Lijun Mou, Wenjing Su, Xin Chen, Ting Zhang, Yifan Xie, Jing Xue, Pui Y. Lee, Huaxiang Wu, Yan Du

**Affiliations:** ^1^ Department of Rheumatology, The Second Affiliated Hospital of Zhejiang University School of Medicine, Hangzhou, China; ^2^ Department of Nephrology, The Second Affiliated Hospital of Zhejiang University School of Medicine, Hangzhou, China; ^3^ Department of Pathology, Shandong Provincial Hospital affiliated to Shandong First Medical University, Jinan, China; ^4^ Division of Immunology, Boston Children’s Hospital, Harvard Medical School, Boston, MA, United States

**Keywords:** lupus nephritis, programmed cell death, ferroptosis, CD163, PC

## Abstract

**Background:**

Dysregulation of cell death and defective clearance of dying cells are closely related to the pathogenesis of lupus nephritis (LN). However, the contribution of a recently discovered form of programmed cell death (PCD) called ferroptosis to LN has not been explored in detail. The purpose of this study was to investigate the role of ferroptosis and its associated metabolic pathways in the pathogenesis of LN.

**Methods:**

The composite gene expression scores were calculated by averaging the z-scored transformed log2 expressed genes within each form of PCD and pathway. Immunohistochemistry and immunofluorescence assays were used to verify the bioinformatics results.

**Results:**

We determined that ferroptosis is prominently and specifically elevated in the glomerular compartment of LN patients compared to other forms of PCD and kidney disease. This finding was then verified by immunohistochemical staining of 4-HNE (a key indicator for ferroptosis) expression in our own cohort (P < 0.0001). Intercorrelation networks were observed between 4-HNE and blood urea nitrogen, SLE disease activity index, serum creatinine, and complement 4, and negatively correlated with glomerular filtration rate in our own LN cohort (P < 0.05). Furthermore, enhanced iron metabolism and reduced fatty acid synthesis may be the most important factors for ferroptosis within the glomerulus. Through analysis of a single cell sequencing dataset and verification of immunohistochemical and immunofluorescence staining, aberrantly activated lipid peroxidation in CD163+ macrophages and CD10+ PC+ (pyruvate carboxylase) epithelial cells indicated that they may be undergoing ferroptosis in the glomerular compartment.

**Conclusions:**

Two dysregulated genes, CD163 and PC, were identified and verified that were significantly associated with lipid peroxidation. Targeting ferroptosis in CD163+ macrophages and CD10+ PC+ epithelial cells may provide novel therapeutic approaches in LN.

## 1 Introduction

Lupus nephritis (LN) is one of the most common complications of systemic lupus erythematosus (SLE) associated with high morbidity and mortality (1). Despite the progress in understanding the pathogenesis of LN, treatment options are still limited. Dysregulation of cell death and defective clearance of dying cells lead to the production of autoantigens, induction of autoantibodies, and deposition of circulating immune complexes in patients with LN (2). Multiple types of programmed cell death (PCD), like apoptosis, pyroptosis, autophagy, and necroptosis, are prominently involved in the pathogenesis of LN (2, 3). However, the contribution of a recently discovered type of PCD, that is, ferroptosis, to LN has not been studied in detail (4).

Unlike other types of PCD, ferroptosis depends on lipid peroxidation and iron overload. Several studies have reported that ferroptosis is involved in the pathogenesis and progression of autoimmune diseases, such as rheumatoid arthritis and SLE (5–8). Li et al. (7) reported that neutrophil ferroptosis is an important driver of SLE neutropenia and contributes significantly to the disease presentation. In addition, ferroptosis may promote the development of SLE by altering Th1/Th2 ratio (8). They all indicated that inhibition of ferroptosis reduced the production of autoantibodies and various inflammatory cytokines, and alleviated the severity of lupus nephritis in lupus-prone mice. Therefore, it is necessary to explore the role of ferroptosis in LN thoroughly and clarify which renal cells undergo ferroptosis.

In this work, we collected and analyzed microarray data from the Gene Expression Omnibus (GEO) database of kidney tissue from LN patients and living donors, as well as expression data from single-cell sequencing and the Nephroseq database. By calculating the composite gene expression scores by averaging z-scored transformed log2 expressed genes within the pathway (9), we determined the role of ferroptosis and its associated metabolic pathways in the pathogenesis of LN.

## 2 Materials and methods

### 2.1 Patient samples and informed consent

A total of 46 renal biopsy tissues used for histological staining were collected from patients with clinically diagnosed SLE and active LN at the Second Affiliated Hospital of Zhejiang University School of Medicine from June 2020 to October 2021. Ethical approval was obtained from the Ethics Committee of the Second Affiliated Hospital of Zhejiang University School of Medicine, Hangzhou, China (approval number: 2020-306). Seven paracancer tissues (considered as controls) were obtained from the Department of Pathology, Shandong Provincial Hospital affiliated with Shandong First Medical University, from October 2021 to February 2022. Ethical approval was obtained from the Ethics Committee of the Shandong Provincial Hospital affiliated with Shandong First Medical University, Jinan, China (approval number: SWYX: NO. 2021-277). All SLE patients met the American College of Rheumatology 1997 criteria and the Systemic Lupus International Collaborating Clinics 2012 criteria for SLE (10, 11). LN was classified according to the 2003 International Society of Nephrology/Renal Pathology Society consensus (12). The clinical characteristics of the 46 LN patients and seven living donors (LDs) are shown in **Supplementary Table 1** (all the lab data from urine and serum were obtained at the time of kidney biopsy).

### 2.2 Immunohistochemistry and immunofluorescence

Following sample collection, the tissue specimens were quickly and thoroughly fixed in 4% paraformaldehyde (P1110; Solarbio, Beijing, China) or frozen at −80°C. After the tissues were dehydrated and made transparent, paraffin (preheated to 60°C) was added and left overnight. The fixed or frozen tissues were then sectioned using a microtome, and the paraffin-fixed tissues were subjected to antigen retrieval. All tissue sections were treated with 5% bovine serum albumin (BSA) and hydrogen peroxide to block endogenous enzyme activity, washed, and incubated with a primary antibody working solution overnight at 4°C. For immunohistochemical staining, the sections were incubated with an appropriate amount of biotin-labeled secondary antibody for 30 min at room temperature. After the DBA chromogenic agent was added for 5–10 min, the sections were rinsed, redyed, dehydrated, made transparent, sealed, and observed under a forward microscope (Leica DM3000 LED, Wetzlar and Mannheim, Germany). For tissue immunofluorescence staining, the sections were incubated with an appropriate amount of fluorescein-conjugated secondary antibody for 1 h at room temperature. After being rinsed with phosphate-buffered saline (PBS), the sections were redyed, sealed, and observed with a forward fluorescence microscope (Leica DM6B, Wetzlar and Mannheim, Germany). For cell immunofluorescence staining, cell slides were fixed with 4% paraformaldehyde (P1110; Solarbio) and sealed with 5% BSA. After incubation with corresponding primary and secondary antibodies, the cells were observed under a forward fluorescence microscope (Leica DM6B). The antibodies used for the immunostaining were anti-4-HNE (ab46545; Abcam, Cambridge, UK), anti-CD163 (YM6146; ImmunoWay, Plano, TX, USA), anti-pyruvate carboxylase (anti-PC, 16588-1-AP, ProteinTech, Chicago, IL, USA), anti-pyruvate carboxylase (sc-271493, Santa Cruz Biotechnology, Dallas, TX, USA), anti-ATP6V0A4 (DF14858, Affinity Biosciences, Cincinnati, OH, USA), Dylight 488 Goat Anti-Rabbit IgG (H+L) (A23220; Abbkine, Wuhan, China), and Dylight 594 Goat Anti-Mouse IgG (H+L) (A23410; Abbkine).

### 2.3 Calculation of immunohistochemistry score and cell counts for immunofluorescence

Under high-magnification imaging, three fields of whole renal tissue and glomerulus or tubules were selected, and the mean value of the area of positive staining for 4-HNE, CD163, pyruvate carboxylase (PC), and ATP6V0A4 was calculated by ImageJ software (National Institutes of Health, Bethesda, MD, USA) and IHC Toolbox plugin (https://imagej.nih.gov/ij/plugins/ihc-toolbox/index.html). Cell counts for the immunofluorescence experiment were calculated by ImageJ software under high-magnification imaging.

### 2.4 Acquisition of mRNA microarray expression data

We searched the Gene Expression Omnibus database (https://www.ncbi.nlm.nih.gov/geo/) for mRNA microarray expression data in LN. We then used the following screening criteria to select mRNA datasets: 1) tissues from renal biopsies of LN patients and LDs and 2) >5 healthy and LN samples. We found and downloaded four mRNA datasets (GSE112943, GSE104948, GSE104954, and GSE32591) that met these criteria. The basic information for the microarray datasets is shown in **Supplementary Table 2**.

### 2.5 Data normalization and identification of differentially expressed genes

The raw data for the datasets were downloaded from the GEO database. The affy package in R software (version 4.0.1) was used to preprocess and normalize the data using the Robust Multiarray Average method (13), the Limma package was used to conduct gene analysis of inter-sample differences, and multiple hypothesis testing and correction were conducted after the *p*-value was obtained. The threshold *p*-value was determined by controlling the false discovery rate, and the corrected *p*-value was the adjusted *p*-value (14, 15). The screening criteria were log2 (fold change) >1 or <−1 and adjusted *p*-value <0.05.

### 2.6 Selected programmed cell death and ferroptosis-related metabolic genes

First, the hallmark, Gene Ontology (GO), Kyoto Encyclopedia of Genes and Genomes (KEGG), REACTOME, and WikiPathways gene sets were downloaded from the Gene Set Enrichment Analysis (GSEA) and molecular characteristic database (MSigDB) website (http://www.gsea-msigdb.org/gsea/index.jsp). Next, gene sets associated with seven types of PCD (apoptosis, ferroptosis, pyroptosis, autophagy, necroptosis, paraptosis, and cuproptosis) and eight ferroptosis-related metabolic pathways (fatty acid biosynthesis, glutathione (GSH) synthesis, iron metabolism, oxidative phosphorylation, glycolysis, tricarboxylic acid (TCA) cycle, glutamine metabolism, and selenium metabolism) were extracted (**Supplementary Tables 3**, **4**). The ferroptosis gene list (39 genes) was extracted from the WP_FERROPTOSIS gene set. The apoptosis gene list (32 genes) was obtained as the intersection of the HALLMARK_APOPTOSIS and REACTOME_APOPTOSIS gene sets. The autophagy gene list (28 genes) was obtained as the intersection of the WP_AUTOPHAGY and REACTOME_AUTOPHAGY gene sets. The necroptosis gene list (22 genes) was obtained as the intersection of the KEGG database and REACTOME_REGULATED_NECROSIS gene sets. The pyroptosis gene list (32 genes) was extracted from the study by Ye et al. (16). The paraptosis gene list (12 genes) was obtained by searching for the keywords “paraptosis” and related literature (17, 18). The cuproptosis gene list (10 genes) was extracted from the study by Tsvetkov et al. (19). The fatty acid biosynthesis gene list (22 genes) and glutathione synthesis gene list (16 genes) were from the PathCards database (https://pathcards.genecards.org/). The iron metabolism gene list (70 genes) was extracted from the study by Li et al. (20). The oxidative phosphorylation gene list (83 genes) was extracted from the KEGG_OXIDATIVE_PHOSPHORYLATION gene set. The glycolysis gene list (29 genes) was extracted from the study by Grayson et al. (9). The TCA cycle gene list (23 genes) was obtained as the intersection of the PathCards_TCA cycle III (animals) and KEGG_CITRATE_CYCLE_TCA_CYCLE gene sets. The glutamine metabolism gene list (23 genes) was extracted from the GOBP_GLUTAMINE_METABOLIC_PROCESS gene set. The selenium metabolism gene set (25 genes) was extracted from the study by Kryukov et al. (21).

### 2.7 Calculation of the composite gene expression scores

The composite gene expression scores were calculated by averaging the z-scored transformed log2 expressed genes within each form of PCD and pathway (9).

### 2.8 Analysis of CD163, PC, and ATP6V0A4 expression features in LN based on the Nephroseq database

Nephroseq (https://nephroseq.org/) is a web-based analysis engine for studies on kidney diseases and related disorders. CD163, PC, and ATP6V0A4 mRNA expression levels according to LN histological classes (GSE32591 cohort: 8 class II, 8 class III, 13 class IV, and 2 class V) separated into the tubulointerstitial and glomerular compartments were extracted from the Nephroseq database.

### 2.9 Statistical analysis

IBM SPSS Statistics 25 (IBM Corp.) and GraphPad Prism 8.0 (GraphPad Software Inc.) were used to analyze the data and draw scatter diagrams. Normally distributed data were expressed as mean ± standard deviation, and the differences between the groups were analyzed by Student’s t-test. Non-parametric data were expressed as median (range), and differences between groups were analyzed by the Mann–Whitney U test. Spearman’s correlation coefficient analysis was applied to detect correlations between the two groups. Values of *p* < 0.05 were considered significant. The numbers of independent technical repeats (*n*) are indicated in the figure legends.

## 3 Results

### 3.1 Alteration of seven types of PCD in living donors and lupus nephritis patients

**Figure 1:**
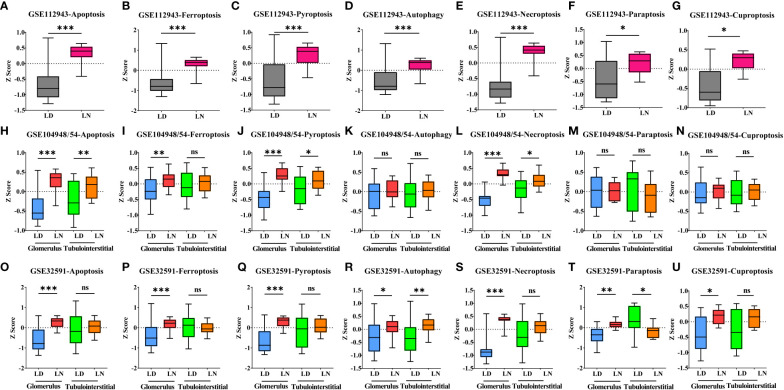
Alteration of seven types of PCD in living donors and lupus nephritis patients. **(A–G)**. Bar graph of apoptosis **(A)**, ferroptosis **(B)**, pyroptosis **(C)**, autophagy **(D)**, necroptosis **(E)**, paraptosis **(F)**, and cuproptosis **(G)** gene expression within the whole kidney in the GSE112943 cohort. **(H–N)** Bar graph of apoptosis **(H)**, ferroptosis **(I)**, pyroptosis **(J)**, autophagy **(K)**, necroptosis **(L)**, paraptosis **(M)**, and cuproptosis **(N)** gene expression within the glomerular and tubulointerstitial compartments in the GSE104948/54 cohort. **(O–U)** Bar graph of apoptosis **(O)**, ferroptosis **(P)**, pyroptosis **(Q)**, autophagy **(R)**, necroptosis **(S)**, paraptosis **(T)**, and cuproptosis **(U)** gene expression within the glomerular and tubulointerstitial compartments in the GSE32591 cohort. *p < 0.05; **p < 0.01; ***p < 0.001; ns, not significant. Values of *p* < 0.05 were considered significant. PCD, programmed cell death.

Gene expression signatures for seven types of PCD were profiled in the whole kidneys or the glomerular and tubulointerstitial compartments between patients with LN and LD. In the whole kidney tissues (GSE112943 cohorts), it was found that seven types of PCD were all significantly increased in 14 LN patients compared with seven LDs (**Figures 1A–G**, *p* < 0.05). This is in line with previous studies (2). In the glomerular compartment of the GSE104948 and GSE32591 cohorts, the gene expression of ferroptosis, apoptosis, pyroptosis, and necroptosis was significantly increased in two independent datasets in patients with LN relative to LD (*p* < 0.05), while the gene expression of autophagy, paraptosis, and cuproptosis was only significantly increased (*p* < 0.005) in the GSE32591 cohort and had no significance in the GSE104948 cohort. However, different patterns were observed in the tubulointerstitial compartment. Compared with LD, the gene expression of apoptosis, pyroptosis, and necroptosis was only significantly increased (*p* < 0.05) in patients with LN from the GSE32591 cohort, while the gene expression of autophagy was only significantly increased (*p* = 0.0074) in patients with LN from the GSE104954 cohort. In contrast, there was significantly decreased gene expression of the paraptosis in patients with LN relative to LD (*p* = 0.0281). Additionally, gene expression related to ferroptosis and cuproptosis was not statistically significant in patients with LN compared with LD (**Figures 1B–U**). These results suggest that ferroptosis is prominently and specifically elevated in the glomerular compartment of LN patients from two independent cohorts compared to other forms of PCD.

### 3.2 Level of ferroptosis in LN and other kidney diseases and association with kidney function

**Figure 2:**
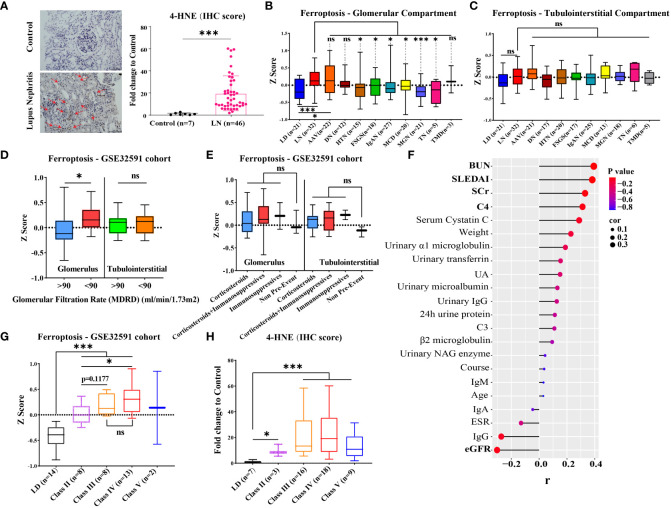
Level of ferroptosis in LN and other kidney diseases and association with kidney function. **(A)** Representative immunohistochemistry images of 4-HNE in 46 LN patients and seven LDs. The red arrows indicate 4-HNE-positive regions. **(B, C)** Gene expression of ferroptosis within the glomerular and tubulointerstitial compartments of various renal diseases and living donors in GSE104948/54 cohort. **(D)** Gene expression of ferroptosis within the glomerular and tubulointerstitial compartments in distinguishing LN patients (GSE32591 cohort) with GFR ≥ 90 from those with GFR < 90. **(E)** Gene expression of ferroptosis within the glomerular and tubulointerstitial compartments in LN patients (GSE32591 cohort) with treatment by concomitant glucocorticoid and/or immunosuppressants. **(F)** Correlation of 4-HNE level and clinical features of 46 LN patients in our own LN cohort. **(G, H)** Gene expression of ferroptosis within the glomerular compartments in different classes of LN patients from GSE32591 cohort **(G)** and our own LN cohort **(H)**. LD, living donors; LN, lupus nephritis; AAV, antineutrophil cytoplasmic antibody-associated vasculitis; DN, diabetic nephropathy; HTN, hypertensive nephropathy; IgAN, IgA nephropathy; FSGS, focal segmental glomerulosclerosis; TMD, thin membrane disease; MCD, minimal change disease; MGN, membranous glomerulonephropathy; TN, tumor nephrectomy; IHC, immunohistochemical; SLEDAI, systemic lupus erythematosus disease activity index; ESR, erythrocyte sedimentation rate; C3/C4, complement 3/4; eGFR, estimated glomerular filtration rate; SCr, serum creatinine; BUN, blood urea nitrogen; UA, uric acid. *p < 0.05; ***p < 0.001; ns, not significant. Values of *p* < 0.05 were considered significant.

Immunohistochemical staining revealed that the expression of 4-HNE (lipid peroxidation), a key indicator for ferroptosis, was significantly increased in the glomerulus and tubules of 46 LN patients compared with seven controls (**Figure 2A**, *p* < 0.001). To confirm if the increased ferroptosis level was unique to LN, we compared the gene expression of ferroptosis for various renal diseases in the GSE104948 cohort (**Figures 2B, C**). The gene expression of ferroptosis did not differ between LD, normal kidney biopsy sections from patients who underwent tumor nephrectomy and patients with focal segmental glomerulosclerosis (FSGS), IgA nephropathy (IgAN), minimal change disease (MCD), and membranous glomerulonephropathy (MGN). The gene expression of ferroptosis in LN was significantly greater than in LD (*p* < 0.001) but was not different from antineutrophil cytoplasmic antibody-associated vasculitis (AAV). Interestingly, compared with kidney from the four forms of nephrotic syndrome with potential inflammatory components (FSGS, IgAN, MCD, MGN) and also the hypertensive nephropathy and tumor nephrectomy, the gene expression of ferroptosis was significantly increased in the glomerular compartment of LN (*p* < 0.05). In an analysis restricted to patients with LN in the Berthier Lupus cohort (GSE32591) where detailed clinical features were available for review, the gene expression of ferroptosis was increased in the glomerular compartment of patients with LN that estimated glomerular filtration rate (eGFR) less than 90 ml/min/1.73 m^2^ (**Figure 2D**). However, there was no significant difference in ferroptosis score between patients categorized by concomitant glucocorticoid and/or immunosuppressants used at the time of biopsy and those not treated (**Figure 2E**). Similarly, 4-HNE levels were positively correlated with blood urea nitrogen, SLE disease activity index, serum creatinine, and complement 4 but negatively correlated with eGFR in our own LN cohort (**Figure 2F**, *p* < 0.05). In addition, ferroptosis levels were higher in LN patients at class III and IV compared with LD and other classes in both the GSE32591 cohort and our own LN cohort (**Figures 2G, H**).

### 3.3 Alteration of ferroptosis-related metabolic pathways in the glomerular and tubulointerstitial compartments

**Figure 3:**
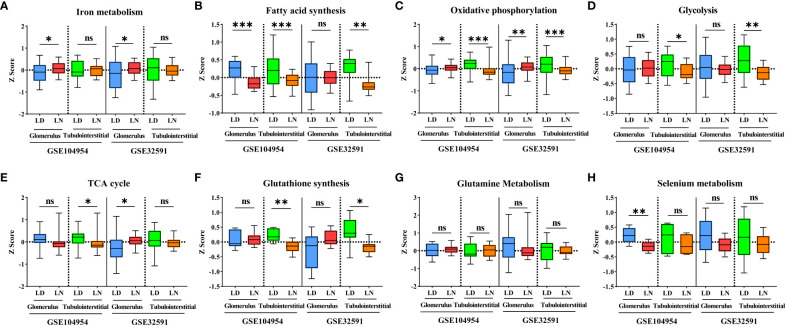
Alteration of eight ferroptosis-related metabolic pathways in LN patients. **(A–H)** Bar graphs for gene expression of iron metabolism **(A)**, fatty acid synthesis **(B)**, oxidative phosphorylation **(C)**, glycolysis **(D)**, TCA cycle **(E)**, glutathione synthesis **(F)**, glutamine metabolism **(G)**, and selenium metabolism **(H)** pathways within the glomerular and tubulointerstitial compartments in GSE104948/54 and GSE32591 cohorts. *p < 0.05; **p < 0.01; ***p < 0.001; ns, not significant. Values of *p* < 0.05 were considered significant. LN, lupus nephritis; TCA, tricarboxylic acid.

**Figure 4:**
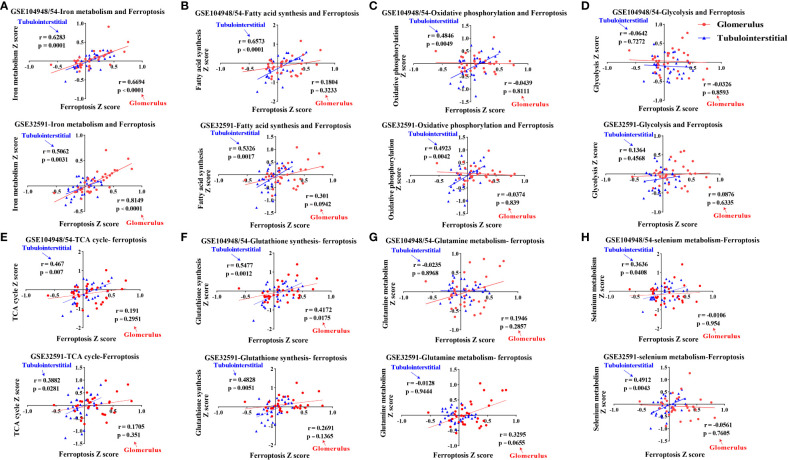
Correlations of eight ferroptosis-related metabolic pathways and ferroptosis in LN patients. **(A–H)** Correlations of iron metabolism **(A)**, fatty acid synthesis **(B)**, oxidative phosphorylation **(C)**, glycolysis **(D)**, TCA cycle **(E)**, glutathione synthesis **(F)**, glutamine metabolism **(G)**, and selenium metabolism **(H)** pathways with ferroptosis within the glomerular and tubulointerstitial compartments in GSE104948/54 and GSE32591 cohort. Values of *p* < 0.05 were considered significant. LN, lupus nephritis; TCA, tricarboxylic acid.

Ferroptosis is associated with multiple metabolic pathways. Therefore, we further investigated the gene signatures for eight major ferroptosis-related metabolic pathways in the glomerular and tubulointerstitial compartments between patients with LN and LD (**Figure 3**). In the glomerular compartment of the GSE104948 and GSE32591 cohorts, the gene expression of iron metabolism and oxidative phosphorylation was significantly increased in two independent datasets in patients with LN relative to LD (*p* < 0.05), while the gene expression of fatty acid synthesis was significantly downregulated (*p* < 0.05) in GSE104948 cohort. In the tubulointerstitial compartment, the gene expression of fatty acid synthesis, oxidative phosphorylation, glycolysis, and GSH synthesis was significantly downregulated (*p* < 0.05). However, the TCA cycle, glutamine metabolism, and selenium metabolism have no consistent significance in the glomerular and tubulointerstitial compartments of the GSE104948 and GSE32591 cohorts. There was a strong, positive correlation (*p* < 0.01) between the ferroptosis and iron metabolism in the glomerular compartment of the GSE104948 and GSE32591 cohorts, while the ferroptosis was positively correlated (*p* < 0.05) with iron metabolism, fatty acid synthesis, oxidative phosphorylation, TCA cycle, and selenium metabolism in the tubulointerstitial compartment (**Figure 4**).

### 3.4 Dysregulation of CD163, PC, and ATP6V0A4 in the glomerular compartment

**Figure 5:**
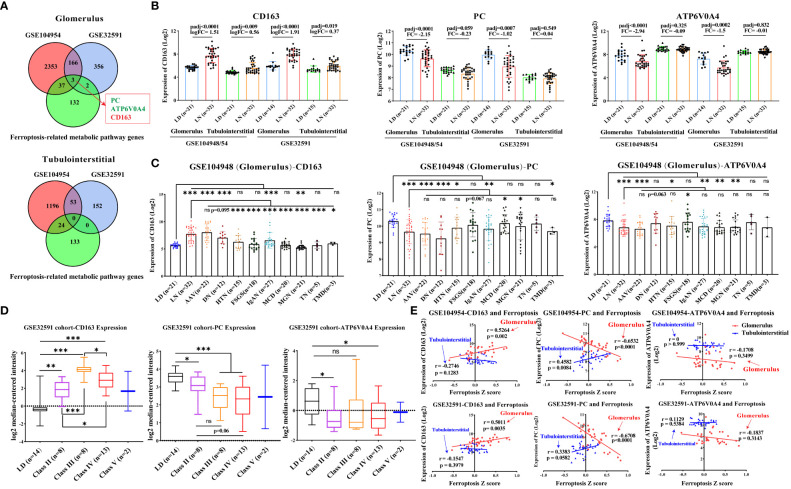
Dysregulation of CD163, PC, and ATP6V0A4 in the glomerular compartment of LN patients. **(A)** Venn diagram shows the intersection of differentially expressed genes and metabolism-related genes in the glomerular and tubulointerstitial compartments. **(B)** Expression of CD163, PC, and ATP6V0A4 in the GSE104948/54 and GSE32591 cohorts. **(C)** Expression of CD163, PC, and ATP6V0A4 within the glomerular compartments of various renal diseases and living donors in the GSE104948 cohort. **(D)** Expression of CD163, PC, and ATP6V0A4 within the glomerular compartments in different classes of LN patients from GSE32591 cohort. **(E)** Correlation of expression of CD163, PC, and ATP6V0A4 and ferroptosis within the glomerular and tubulointerstitial compartments in GSE104948/54 and GSE32591 cohorts. LD, living donors; LN, lupus nephritis; AAV, antineutrophil cytoplasmic antibody-associated vasculitis; DN, diabetic nephropathy; HTN, hypertensive nephropathy; IgAN, IgA nephropathy; FSGS, focal segmental glomerulosclerosis; TMD, thin membrane disease; MCD, minimal change disease; MGN, membranous glomerulonephropathy; TN, tumor nephrectomy; PC, pyruvate carboxylase. *p < 0.05; **p < 0.01; ***p < 0.001; ns, not significant. Values of *p* < 0.05 were considered significant.

To find more accurate targets regulating ferroptosis, we examined the intersection of differentially expressed genes in the GSE104948 and GSE32591 cohorts and metabolism-related genes. One upregulated gene (CD163) and two downregulated genes (PC and ATP6V0A4) were identified in the glomerulus compartment (**Figures 5A, B**). Consistent with the gene expression of ferroptosis, dysregulation of CD163, PC, and ATP6V0A4 was specific in the glomerular compartment of patients with LN from the GSE104948 cohort compared to other kidney diseases (especially FSGS, MCD, and MGN) (**Figure 5C**). Meanwhile, CD163 and PC expression was more dysregulated in LN patients at stages III and IV compared with that in LD and other stages in the GSE32591 cohort, while ATP6V0A4 expression was only downregulated in LN patients at stages II and IV compared with LD (**Figure 5D**). There was a strong, positive correlation (*p* < 0.01) between CD163 and ferroptosis in the glomerular compartment of the GSE104948 and GSE32591 cohorts, while a strong, negative correlation (*p* < 0.0001) between PC and ferroptosis. In addition, ATP6V0A4 was negatively associated with ferroptosis in the glomerular compartment, but there was no significant (**Figure 5E**).

### 3.5 Ferroptosis of CD163^+^ macrophages and CD10^+^ PC^+^ epithelial cells in the glomerular compartment

**Figure 6:**
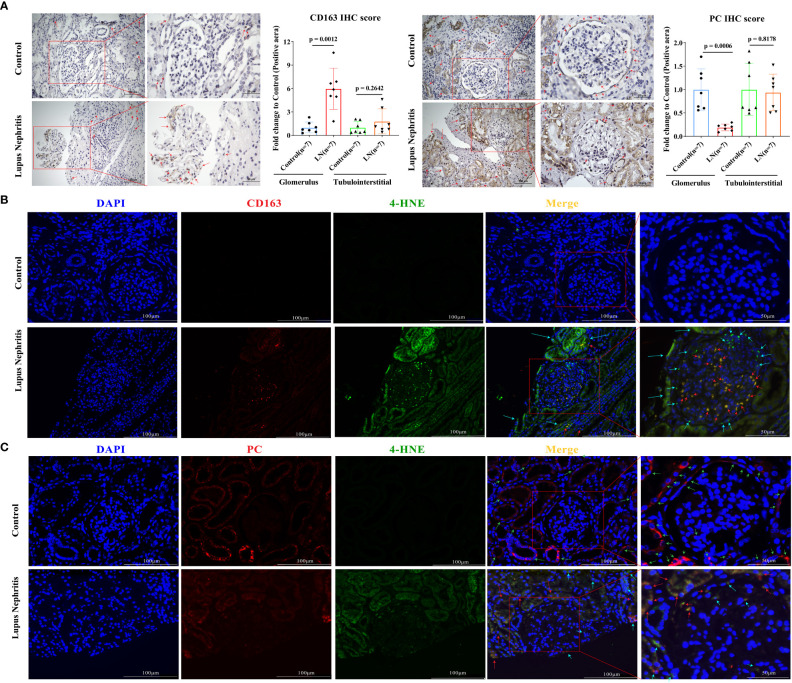
Ferroptosis of CD163^+^ macrophages and CD10^+^ PC^+^ epithelial cells in the glomerulus of LN patients. **(A)** Representative immunohistochemistry images of CD163 and PC in seven LN patients and seven controls. The red arrows indicate positive regions. **(B)** Representative immunofluorescent staining images demonstrate co-localization of 4-HNE (green) and CD163 (red) within macrophages in the glomerular compartment. **(C)** Representative immunofluorescent staining images demonstrate co-localization of 4-HNE (green) and PC (red) within epithelial cells in the glomerular compartment. The red arrows indicate cells double-positive for CD163 or PC and 4-HNE that were undergoing ferroptosis; green arrows indicate cells PC-positive cells or CD163-positive cells; sky-blue arrows indicate 4-HNE-positive cells. Values of *p* < 0.05 were considered significant. LN, lupus nephritis; PC, pyruvate carboxylase.

In our own LN cohort, an immunohistochemical staining assay verified that the level of CD163 was significantly increased in the glomerulus of LN patients compared with controls (**Figure 6A**, *p* < 0.001), while PC expression was significantly reduced. However, in the tubulointerstitial compartment, there was no difference in CD163 and PC levels between LN patients and controls. Unfortunately, the level of ATP6V0A4 had no significance in the glomerulus and tubulointerstitial compartments of LN patients and controls (**Figure 6A**; **Supplementary Figure 1**). Next, analysis of a single-cell sequencing dataset (ImmPort ID: SDY997) from Accelerating Medicines Partnership Phase I project indicated that CD163 was mainly expressed in macrophages (Cluster Macrophage (CM)1, CM2, CM3, and CM4), PC was mainly expressed in CD10^+^ epithelial cells (CE0), while ATP6V0A4 is only slightly expressed in B cells, T cells, CE0, CM1, and dividing cells (**Supplementary Figure 2**). Importantly, immunofluorescence staining revealed that CD163 and 4-HNE were mainly co-localized in the glomerulus of LN patients (**Figure 6B**; **Supplementary Figures 3A–C**). Moreover, PC was co-localized with 4-HNE in the glomerulus of LN patients (**Figure 6C**; **Supplementary Figures 3D–F**). These results suggested that lipid peroxidation of CD163^+^ macrophages and CD10^+^ PC^+^ epithelial cells in the glomerular compartment were aberrantly activated, and they may undergo ferroptosis.

## 4 Discussion

The high heterogeneity of SLE or LN patients leads to great challenges in its diagnosis and treatment. Precisely because of this, the exact pathogenesis of LN remains unclear. Ferroptosis, characterized by lipid peroxidation and iron overload, is associated with the alteration of multiple metabolic pathways, such as iron metabolism, glycometabolism, and lipid metabolism (22). Recently, several studies have reported that many ferroptosis-related genes were related to immune-infiltrated cells (especially monocyte) in LN through analysis of a single GEO dataset (23, 24). Additionally, Alli et al. reported that ferroptosis plays an important role in renal tubular damage in LN (25). However, the crosstalk between ferroptosis and metabolic pathways and the relationship between ferroptosis and glomerular injury in LN have not been elucidated.

The present study has revealed the changes in different types of PCD, particularly ferroptosis, which was markedly elevated in LN through analysis of three GEO datasets. By conducting the first in-depth transcriptomic analysis of the changes in various ferroptosis-related metabolic pathways in LN, we provided a new perspective that enhanced iron metabolism and reduced fatty acid synthesis may be significant contributors to ferroptosis within the glomerulus. In addition, higher levels of ferroptosis in LN patients at class III and IV and the strong correlation between ferroptosis and kidney function indicated that ferroptosis was closely correlated with disease activity and progression of LN. Finally, we identified and verified two dysregulated genes, CD163 and PC, that were significantly associated with lipid peroxidation.

CD163 is a scavenger receptor of haptoglobin–hemoglobin complexes and is mainly utilized as a specific marker for macrophages (especially M2-like). It has been reported that CD163 and SLC40A1 were selectively expressed in kidney M2-like macrophages of LN patients, which were closely related to iron homeostasis (26). The rupture of red blood cells caused by SLE-associated autoimmune hemolytic anemia and glomerular injury are the main sources of haptoglobin–hemoglobin complexes. Taken together, these observations suggest that abnormally elevated CD163^+^ tissue-infiltrating macrophages may lead to excessive phagocytosis of haptoglobin–hemoglobin complexes, which may result in excessive accumulation of iron and an increased amount of unstable iron pools within macrophages, leading to oxidative stress and ferroptosis. Elevated CD163 expression may also be an adaptive change in the body to prevent further tissue damage caused by broken red blood cells and their associated complexes. Consistent with this view, treatment with a specific ferroptosis inhibitor was found to ameliorate lupus nephritis and overall disease severity in lupus-prone mice (7). Furthermore, CD163^+^ tissue-infiltrating macrophages were identified as the main infiltrating cellular subgroup in human LN, and urinary soluble CD163 derived from the surface of these cells was closely related to histological inflammation (27). This is consistent with our finding that ferroptosis level and CD163 expression are specifically elevated in LN and AAV. Although both LN patients at stages III and IV and active AAV patients exhibit cellular crescent formation, patients with other types of glomerulonephritis do not. However, studies on the mechanism of CD163^+^ tissue-infiltrating macrophages’ involvement in LN are lacking. Consequently, we believe that ferroptosis is a novel and promising research target.

PC is involved in gluconeogenesis and lipogenesis in the liver and kidney. Accalia Fu et al. (28) reported that PC protects against reactive oxygen species (ROS) accumulation and cell death triggered by inflammation, which is achieved by generating NADPH relevant to thioredoxin and GSH antioxidant pathways (29). Importantly, PC is also essential for the maintenance of GSH pools and GSH/GSSG (oxidized GSH) ratio (28). These findings suggest that PC can be considered a protective factor against ferroptosis. In our study, we found that PC expression and PC-expressing parietal epithelial cells were significantly downregulated in the glomerulus of patients with LN. This further indicated that decreased PC expression, reduced GSH pools, and elevated ROS accumulation in glomerular epithelial cells may contribute to the occurrence of ferroptosis. In addition, due to the possible adjacent epithelial cells’ interaction in the glomerulus, PC-negative cells with ferroptosis may further influence neighboring PC-positive cells to induce ferroptosis. More experiments, both *in vitro* and *in vivo*, are needed to verify this hypothesis. Future research will help us to better understand the mechanism of action in which PC and ferroptosis participate in the pathogenesis of LN.

There are some important potential limitations in this study. First, the main conclusions stated that there was enhanced expression of ferroptosis-related genes in the glomerular rather than tubular compartment of lupus nephritis biopsies compared to living donor controls, in association with increased CD163^+^ cells, and decreased CD10^+^ PC^+^ cells based on analysis of publicly available transcriptomic datasets lacked definitive experimental studies. Second, it has been reported that when some cells undergo cell death, it can induce the activation of a cell death pathway in neighboring cells (2). Whether crosstalk occurs between glomerular macrophage and epithelial cells needs to be confirmed. Additionally, the majority of patients in our cohort were treated with corticosteroids or immunosuppressants at the time of renal biopsy, and treatment may have some effect on the level of ferroptosis (30). Therefore, a larger cohort needs to be recruited for subgroup comparison. Finally, validation of animal models and the association between soluble CD163 in urine and ferroptosis is required.

**Figure 7:**
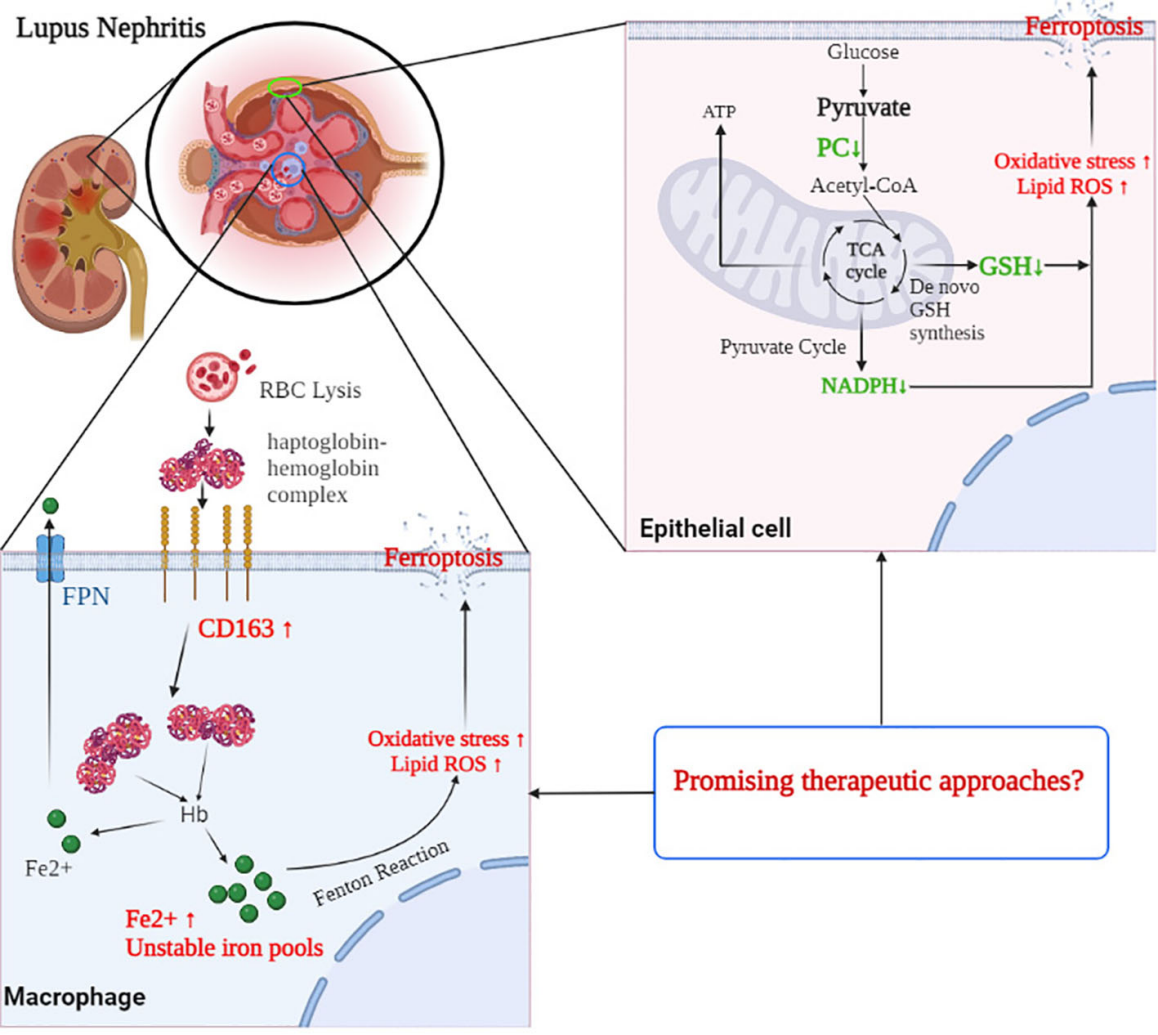
The mechanism of CD163 and PC involved in the process of ferroptosis in the glomerulus of LN patients. CD163 mediated the macrophages taken in haptoglobin–hemoglobin complexes, which come from RBC lysis. However, excessive phagocytosis of haptoglobin–hemoglobin complexes may result in excessive accumulation of iron and an increased amount of unstable iron pools within macrophages, leading to oxidative stress and ferroptosis. In glomerular epithelial cells, decreased PC expression reduced the level of NADPH and GSH pools and elevated the ROS accumulation, which may contribute to the occurrence of ferroptosis. Targeting ferroptosis in CD163^+^ macrophages and PC^+^ epithelial cells may provide promising therapeutic approaches in LN. RBC, red blood cell; NADPH, nicotinamide adenine dinucleotide phosphate; GSH, glutathione; LN, lupus nephritis; PC, pyruvate carboxylase; ROS, reactive oxygen species.

Distinct alterations in programmed cell death and related metabolic pathways were observed in the renal transcriptome from patients with different forms of glomerulonephritis, including systemic inflammatory diseases such as SLE and AAV, four forms of nephrotic syndrome (FSGS, IgAN, MCD, and MGN), and hypertensive and diabetic nephropathy. The overall patterns of gene expression are indicative of increased ferroptosis, iron metabolism, and decreased fatty acid synthesis, especially in the glomerular compartment of LN patients. Aberrantly activated lipid peroxidation in glomerular CD163^+^ macrophages and CD10^+^ PC^+^ epithelial cells were observed in this study, further suggesting that altered ferroptosis and related metabolic pathways may also play a role in the pathophysiology of LN (**Figure 7**). Targeting ferroptosis in CD163^+^ macrophages and CD10^+^ PC^+^ epithelial cells may provide promising therapeutic approaches in LN.

## Data availability statement

The datasets presented in this study can be found in online repositories. The names of the repository/repositories and accession number(s) can be found below: https://www.ncbi.nlm.nih.gov/geo; GSE112943, GSE104948, GSE104954, GSE32591, GSE27045.

## Ethics statement

Our study was approved by the Ethics Committee of the Second Affiliated Hospital of Zhejiang University School of Medicine (approval number: 2020-306) and the Ethics Committee of the Shandong Provincial Hospital affiliated to Shandong First Medical University, Jinan, China (approval number: SWYX: NO.2021-277). All participants signed a written informed consent form. The patients/participants provided their written informed consent to participate in this study.

## Author contributions

QC and YD provided the study concept and design. QC performed most experiments, analyzed the data, and drafted the manuscripts. QC, LM, WS, XC, TZ, JX, YD, and HW contributed to the collection of clinical samples, interpretation of data, and material support. YD and HW contributed to the review of the original manuscript. QC, YX, PL, YD, and HW participated in the revision and review of the revised manuscript. All authors read and approved the final paper.
