# Supplementary material for: Ferroptosis of CD163+ tissue-infiltrating macrophages and CD10+ PC+ epithelial cells in lupus nephritis
Source: Front Immunol. 2023 Jul 31;14:1171318. doi: 10.3389/fimmu.2023.1171318 (PMC10423811; doi:10.3389/fimmu.2023.1171318)
Supplement: Supplementary file 1 [file DataSheet_1.docx]

Supplementary Material

**Supplementary figures**

**
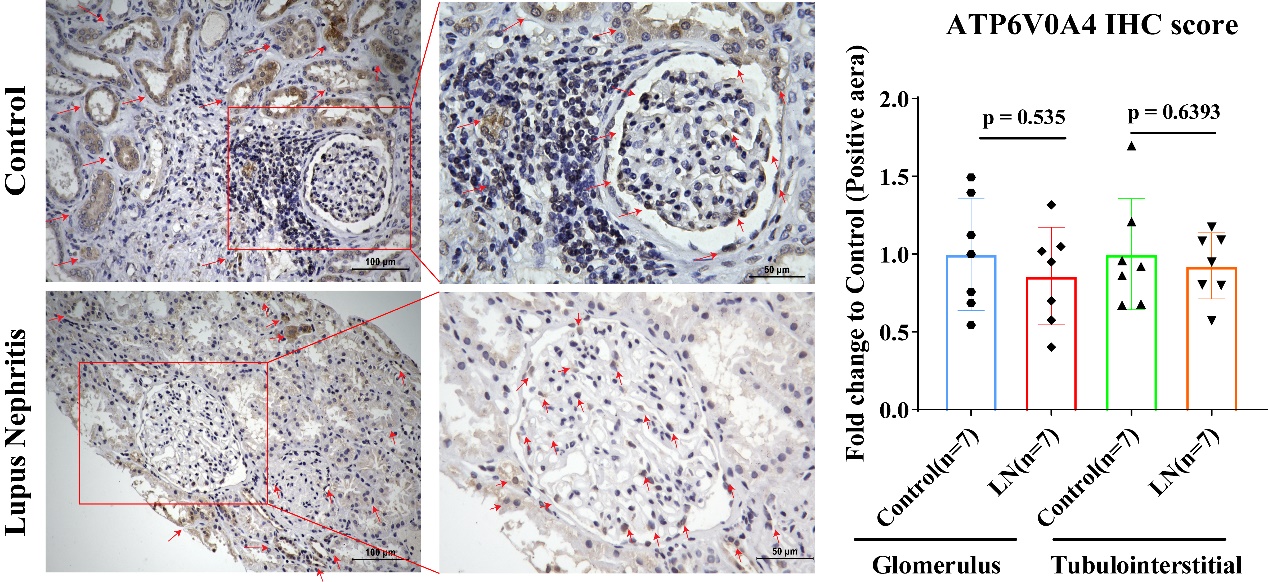
**

**Supplementary Figure 1** **Immunohistochemical staining of ATP6V0A4.** Representative immunohistochemistry images of ATP6V0A4 in 7 LN patients and 7 controls. The red arrows indicate positive regions. Values of *P* < 0.05 were considered significant.

**
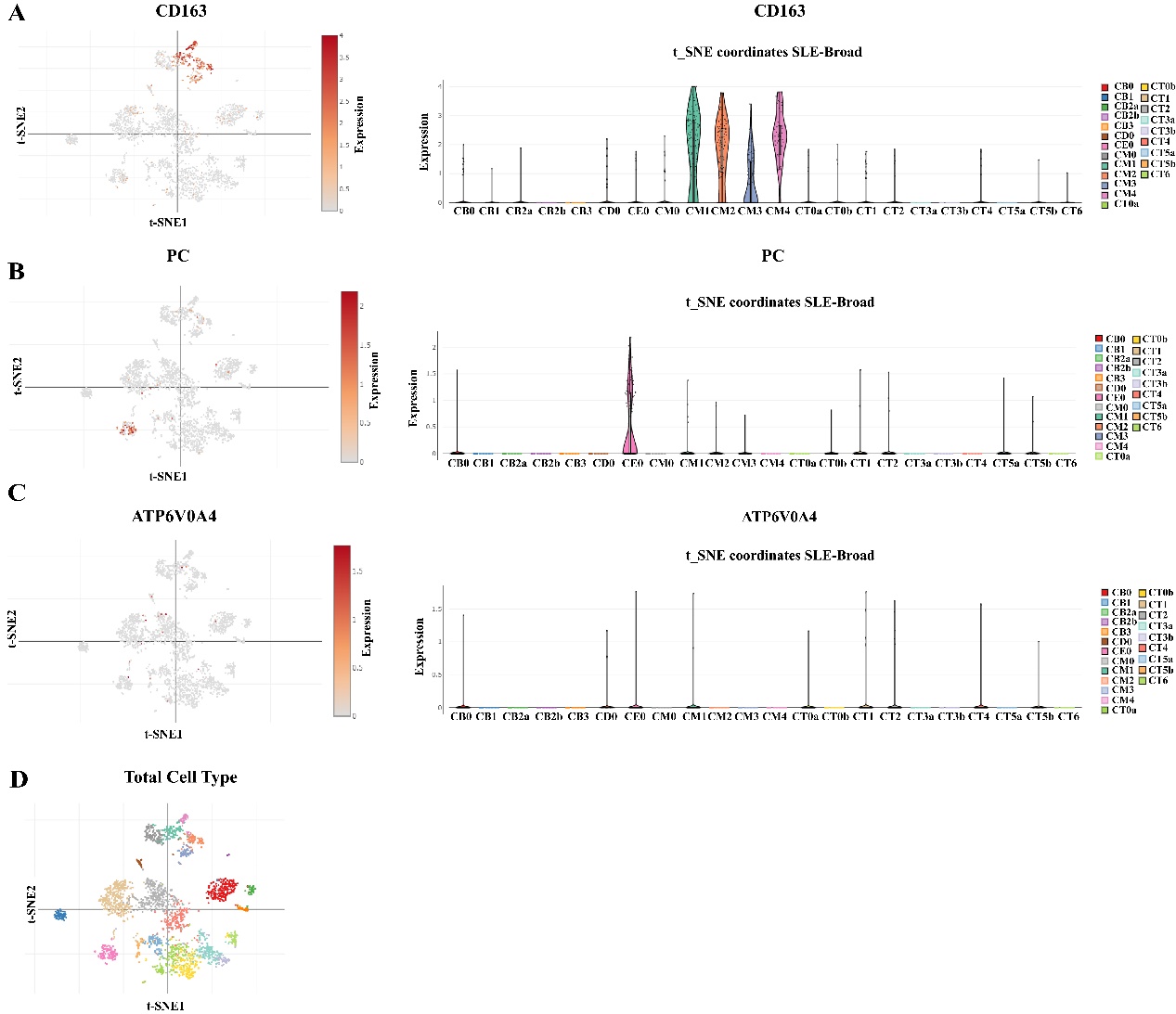
**

**Supplementary Figure 2 Expression of CD163, PC, and ATP6V0A4 in 22 types of cells in a single cell sequencing dataset.** A. Expression of CD163 in 22 types of cells in a single cell sequencing dataset (ImmPort ID: SDY997). B. Expression of PC in 22 types of cells in a single cell sequencing dataset (ImmPort ID: SDY997). C. Expression of ATP6V0A4 in 22 types of cells in a single cell sequencing dataset (ImmPort ID: SDY997). D.t-SNE plot of total cell type. CB, Cluster B cells; CE, Cluster epithelial cells; CM, Cluster Macrophage; CT, Cluster T cells.


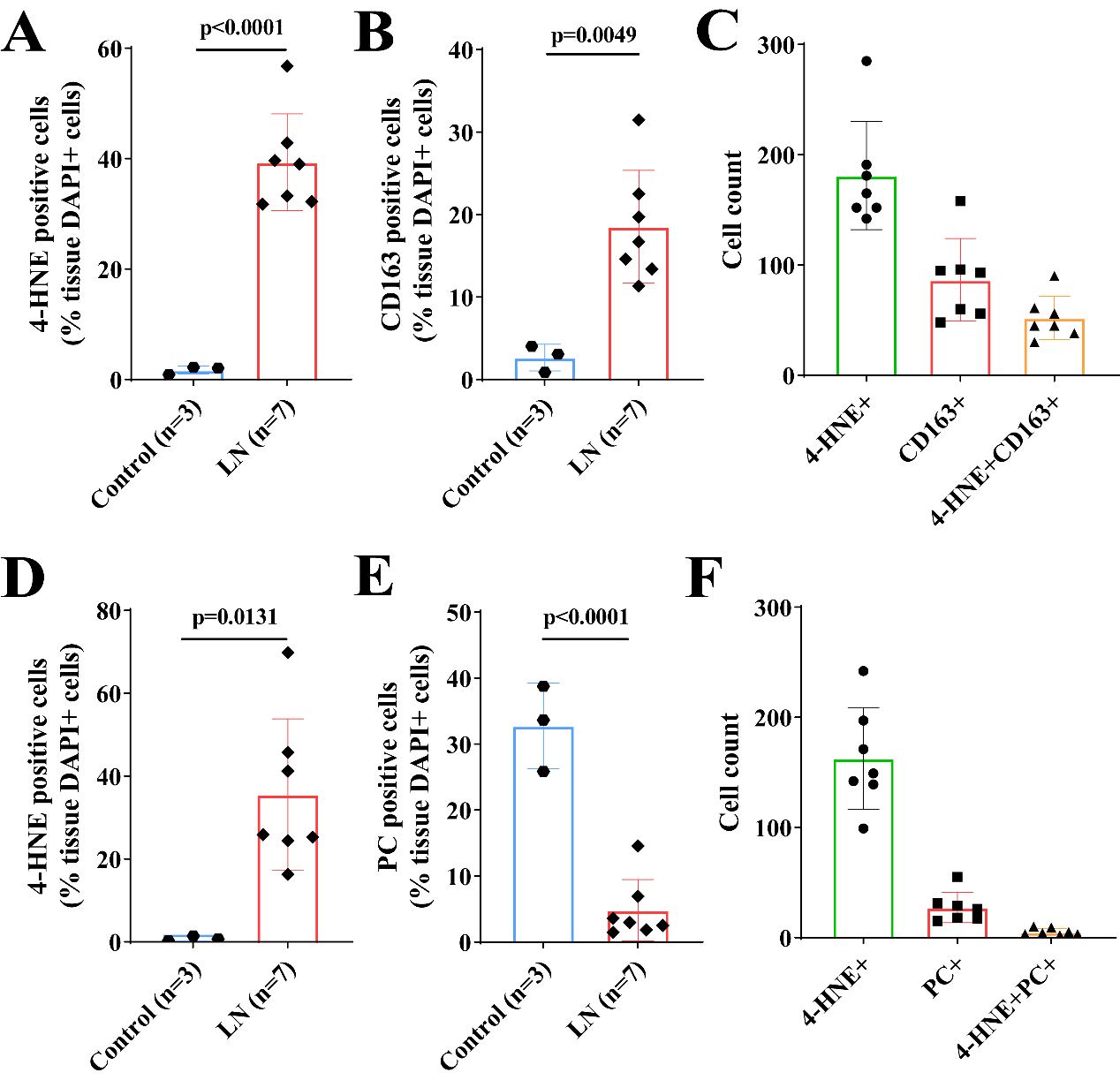


**Supplementary Figure 3** 4-HNE, CD163 and PC expression in kidney tissues from 3 controls and 7 LN patients. A-B. 4-HNE (A) and CD163 (B) expression in total DAPI^+^ cells from 3 controls and 7 LN patients. C. The count of 4-HNE^+^, CD163^+^ and 4-HNE^+^ CD163^+^ cells in 7 LN patients. D-E. 4-HNE (D) and PC (E) expression in total DAPI^+^ cells from 3 controls and 7 LN patients. F. The count of 4-HNE^+^, PC^+^ and 4-HNE^+^ PC^+^ cells in 7 LN patients. Values of *P* < 0.05 were considered significant.

**Supplementary Tables**

**Supplementary Table 1 Clinical characteristics of the 46 LN patients and 7 LDs**

| Items | | **Patients with LN (N=46)** | **Living donors (N=7)** |
| --- | --- | --- | --- |
| **General features** | |  |  |
| Age (Years), mean±SD | | 34.57±11.87 | 45.69±10.23 |
| Sex (Female/male) | | 41/5 | 6/1 |
| Course (Years), median (range) | | 64 (1-263) | N/A |
| SLEDAI, median (range) | | 8.5 (0-18) | N/A |
| 4-HNE IHC score, median (range) | | 10.93 (1.67-103.1) | 1.02 (0.12-2.79) |
| Activity index (AI), median (range) | | 8 (3-24) | N/A |
| Chronic index (CI), median (range) | | 1.5 (0-8) | N/A |
| **Autoantibodies, Positive rate, n (%)** | | | |
| ANA | | 45 (97.8) | N/A |
| Anti-dsDNA | | 26 (56.5) | N/A |
| Anti-SSA | | 23 (50) | N/A |
| Anti-Ro52 | | 19 (41.3) | N/A |
| Anti-SSB | | 5 (10.9) | N/A |
| Anti-Smith | | 9 (19.6) | N/A |
| Anti-RNP | | 24 (52.2) | N/A |
| Anti-RPP | | 18 (39.1) | N/A |
| Anti-cardiolipin | | 4 (8.7) | N/A |
| Anti-nucleosome | | 17 (37) | N/A |
| Anti-histone | | 7 (15.2) | N/A |
| **Laboratory test (normal range), N, median (range)** | | | |
| ESR (0-20mm/h) | | 31 (2-92) | N/A |
| CRP (< 6mg/L) | | 1.95 (0.2-42.2) | N/A |
| IgG (7-16g/L) | | 11.33 (3.41-24.7) | N/A |
| IgA (0.7-4g/L) | | 2.48 (0.43-7.17) | N/A |
| IgM (0.4-2.3g/L) | | 0.97 (0.22-3.66) | N/A |
| C3 (0.82-1.8g/L) | | 0.5 (0.16-1.57) | N/A |
| C4 (100-400mg/L) | | 83.5 (20-360) | N/A |
| C1q (159-233mg/L） | | 139.5 (75-351) | N/A |
| WBC (4-10×10^9^/L) | | 6.25 (2-16.7) | 6.6 (5.4-8.7) |
| Hb (female, 113-151, male, 131-172g/L) | | 113.5 (53-172) | 126 (115-152) |
| PLT (100-300×10^9^/L) | | 203.5 (64-449) | 203.5 (64-449) |
| eGFR (mL/(min×1.73m^2^) | | 91.8 (8.82-199.78) | 114.46 (131.59-158.55) |
| SCr (40-88mmol/L) | | 57.7 (34.9-473.1) | 57.7 (34.9-473.1) |
| BUN (2.8-7.6mmol/L) | | 6.15 (3.1-30.27) | 4.4 (3.2-5) |
| UA (154-357μmol/L) | | 390.5 (186-691) | 209 (138-258) |
| β2-microglobulin (1-3mg/L) | | 3.25 (1.19-33.21) | N/A |
| Serum cystatin C (<1.03mg/L) | | 1.35 (0.58-5.18) | N/A |
| Urinary NAG enzyme (<20U/g·Cr) | | 17.67 (5-47.67) | N/A |
| Urinary α1 microglobulin (<15mg/g·Cr) | | 23.25 (3.14-173.8) | N/A |
| Urinary IgG (<12mg/g·Cr) | | 155.6 (5.63-1333.3) | N/A |
| Urinary transferrin (<2.9mg/g·Cr) | | 146.4 (4.89-660.84) | N/A |
| Urinary microalbumin (<25mg/g·Cr) | | 2267.8 (93.22-8531.5) | N/A |
| 24h urine protein quantification (28-141mg/24h) | | 2206.6 (100.4-12532) | N/A |
| **Medication, Positive rate, n (%)** | |  |  |
| Untreated (first visit) | | 2 (4.4) | N/A |
| Methylprednisolone | <40mg/day | 8 (17.4) | N/A |
|  | ≥40mg/day | 34 (10.9) | N/A |
| Immunosuppressants | Tacrolimus | 5 (10.9) | N/A |
|  | Mycophenolate Mofetil | 5 (10.9) | N/A |
|  | Leflunomide | 1 (2.2) | N/A |
|  | Recombinant human Interleukin-2 | 1 (2.2) | N/A |
|  | Hydroxychloroquine | 15 (32.6) | N/A |

All the lab data from urine and serum were obtained at the time of kidney biopsy. Abbreviations: SLEDAI, systemic lupus erythematosus disease activity index; ESR, erythrocyte sedimentation rate; CRP, C-reactive protein; C3/C4, complement 3/4; WBC, white blood cell; Hb, hemoglobin; PLT, platelet; Treg, regulatory T cells; ANA: antinuclear antibodies; SSA, single-stranded DNA; anti-SSB, anti RNA-protein complex antibodies; RNP, ribonucleoprotein; RPP, Ribosomal P protein; eGFR, Estimated glomerular filtration rate; SCr, serum creatinine; BUN, Blood urea nitrogen; UA, Uric Acid.

**Supplementary Table 2 Basic information of selected GEO datasets**

| GEO accession | Platform | LN tissue category | Diagnosis | |
| --- | --- | --- | --- | --- |
|  |  |  | HC | LN |
| GSE112943 | GPL10558 | Holonephros | 7 | 14 |
| GSE32591 | GPL14663 | Glomerulus and Tubulointerstitial | 14/15 | 32/32 |
| GSE104948 | GPL22945/GPL24120 | Glomerulus | 21 | 32 |
| GSE104954 | GPL22945/GPL24120 | Tubulointerstitial | 21 | 32 |

**Supplementary Table 3 List of genes related to different types of programmed cell death**

| Gene Symbol | Description | Pathway |
| --- | --- | --- |
| AIFM2 | Apoptosis Inducing Factor Mitochondria Associated 2 | Ferroptosis |
| ACSL3 | acyl-CoA synthetase long chain family member 3 | Ferroptosis |
| ACSL4 | acyl-CoA synthetase long chain family member 4 | Ferroptosis |
| ACSL5 | acyl-CoA synthetase long chain family member 5 | Ferroptosis |
| ACSL6 | acyl-CoA synthetase long chain family member 6 | Ferroptosis |
| ALOX15 | arachidonate 15-lipoxygenase | Ferroptosis |
| ATG5 | autophagy related 5 | Ferroptosis |
| ATG7 | autophagy related 7 | Ferroptosis |
| CP | ceruloplasmin | Ferroptosis |
| CYBB | cytochrome b-245 beta chain | Ferroptosis |
| FTH1 | ferritin heavy chain 1 | Ferroptosis |
| FTL | ferritin light chain | Ferroptosis |
| FTMT | ferritin mitochondrial | Ferroptosis |
| GCLC | glutamate-cysteine ligase catalytic subunit | Ferroptosis |
| GCLM | glutamate-cysteine ligase modifier subunit | Ferroptosis |
| GPX4 | glutathione peroxidase 4 | Ferroptosis |
| GSS | glutathione synthetase | Ferroptosis |
| HMOX1 | heme oxygenase 1 | Ferroptosis |
| LPCAT3 | lysophosphatidylcholine acyltransferase 3 | Ferroptosis |
| MAP1LC3A | microtubule associated protein 1 light chain 3 alpha | Ferroptosis |
| MAP1LC3B | microtubule associated protein 1 light chain 3 beta | Ferroptosis |
| MAP1LC3C | microtubule associated protein 1 light chain 3 gamma | Ferroptosis |
| NCOA4 | nuclear receptor coactivator 4 | Ferroptosis |
| PCBP2 | poly(rC) binding protein 2 | Ferroptosis |
| PRNP | prion protein | Ferroptosis |
| SAT1 | spermidine/spermine N1-acetyltransferase 1 | Ferroptosis |
| SAT2 | spermidine/spermine N1-acetyltransferase family member 2 | Ferroptosis |
| SLC11A2 | solute carrier family 11 member 2 | Ferroptosis |
| SLC39A14 | solute carrier family 39 member 14 | Ferroptosis |
| SLC39A8 | solute carrier family 39 member 8 | Ferroptosis |
| SLC3A2 | solute carrier family 3 member 2 | Ferroptosis |
| SLC40A1 | solute carrier family 40 member 1 | Ferroptosis |
| SLC7A11 | solute carrier family 7 member 11 | Ferroptosis |
| STEAP3 | STEAP3 metalloreductase | Ferroptosis |
| TF | transferrin | Ferroptosis |
| TFRC | transferrin receptor | Ferroptosis |
| TP53 | tumor protein p53 | Ferroptosis |
| VDAC2 | voltage dependent anion channel 2 | Ferroptosis |
| VDAC3 | voltage dependent anion channel 3 | Ferroptosis |
| AKT1 | AKT serine/threonine kinase 1 | Apoptosis |
| AKT2 | AKT serine/threonine kinase 2 | Apoptosis |
| AKT3 | AKT serine/threonine kinase 3 | Apoptosis |
| APAF1 | apoptotic peptidase activating factor 1 | Apoptosis |
| BAD | BCL2 associated agonist of cell death | Apoptosis |
| BAX | BCL2 associated X, apoptosis regulator | Apoptosis |
| BCL2 | BCL2 apoptosis regulator | Apoptosis |
| BCL2L1 | BCL2 like 1 | Apoptosis |
| BID | BH3 interacting domain death agonist | Apoptosis |
| BIRC2 | baculoviral IAP repeat containing 2 | Apoptosis |
| CASP3 | caspase 3 | Apoptosis |
| CASP6 | caspase 6 | Apoptosis |
| CASP7 | caspase 7 | Apoptosis |
| CASP8 | caspase 8 | Apoptosis |
| CASP9 | caspase 9 | Apoptosis |
| CFLAR | CASP8 and FADD like apoptosis regulator | Apoptosis |
| CYCS | cytochrome c, somatic | Apoptosis |
| DFFA | DNA fragmentation factor subunit alpha | Apoptosis |
| DFFB | DNA fragmentation factor subunit beta | Apoptosis |
| FADD | Fas associated via death domain | Apoptosis |
| FAS | Fas cell surface death receptor | Apoptosis |
| FASLG | Fas ligand | Apoptosis |
| PPP3CC | protein phosphatase 3 catalytic subunit gamma | Apoptosis |
| PPP3R1 | protein phosphatase 3 regulatory subunit B, alpha | Apoptosis |
| TNFRSF10A | TNF receptor superfamily member 10a | Apoptosis |
| TNFRSF10B | TNF receptor superfamily member 10b | Apoptosis |
| TNFSF10 | TNF superfamily member 10 | Apoptosis |
| TP53 | tumor protein p53 | Apoptosis |
| TRADD | TNFRSF1A associated via death domain | Apoptosis |
| TRAF2 | TNF receptor associated factor 2 | Apoptosis |
| TANK | TRAF family member associated NFKB activator | Apoptosis |
| XIAP | X-linked inhibitor of apoptosis | Apoptosis |
| AMBRA1 | autophagy and beclin 1 regulator 1 | Autophagy |
| ATG101 | autophagy related 101 | Autophagy |
| ATG12 | autophagy related 12 | Autophagy |
| ATG13 | autophagy related 13 | Autophagy |
| ATG14 | autophagy related 14 | Autophagy |
| ATG16L1 | autophagy related 16 like 1 | Autophagy |
| ATG3 | autophagy related 3 | Autophagy |
| ATG5 | autophagy related 5 | Autophagy |
| ATG7 | autophagy related 7 | Autophagy |
| ATG9A | autophagy related 9A | Autophagy |
| BECN1 | beclin 1 | Autophagy |
| MAP1LC3B | microtubule associated protein 1 light chain 3 beta | Autophagy |
| MLST8 | MTOR associated protein, LST8 homolog | Autophagy |
| MTOR | mechanistic target of rapamycin kinase | Autophagy |
| PIK3C3 | phosphatidylinositol 3-kinase catalytic subunit type 3 | Autophagy |
| PIK3R4 | phosphoinositide-3-kinase regulatory subunit 4 | Autophagy |
| PRKAA1 | protein kinase AMP-activated catalytic subunit alpha 1 | Autophagy |
| PRKAA2 | protein kinase AMP-activated catalytic subunit alpha 2 | Autophagy |
| PRKAB1 | protein kinase AMP-activated non-catalytic subunit beta 1 | Autophagy |
| PRKAB2 | protein kinase AMP-activated non-catalytic subunit beta 2 | Autophagy |
| PRKAG1 | protein kinase AMP-activated non-catalytic subunit gamma 1 | Autophagy |
| PRKAG2 | protein kinase AMP-activated non-catalytic subunit gamma 2 | Autophagy |
| PRKAG3 | protein kinase AMP-activated non-catalytic subunit gamma 3 | Autophagy |
| RB1CC1 | RB1 inducible coiled-coil 1 | Autophagy |
| RPTOR | regulatory associated protein of MTOR complex 1 | Autophagy |
| ULK1 | unc-51 like autophagy activating kinase 1 | Autophagy |
| UVRAG | UV radiation resistance associated | Autophagy |
| WIPI2 | WD repeat domain, phosphoinositide interacting 2 | Autophagy |
| AIM2 | absent in melanoma 2 | Pyroptosis |
| CASP1 | caspase 1 | Pyroptosis |
| CASP3 | caspase 3 | Pyroptosis |
| CASP4 | caspase 4 | Pyroptosis |
| CASP5 | caspase 5 | Pyroptosis |
| CASP6 | caspase 6 | Pyroptosis |
| CASP8 | caspase 8 | Pyroptosis |
| ELANE | elastase, neutrophil expressed | Pyroptosis |
| GPX4 | glutathione peroxidase 4 | Pyroptosis |
| GSDMA | gasdermin A | Pyroptosis |
| GSDMB | gasdermin B | Pyroptosis |
| GSDMC | gasdermin C | Pyroptosis |
| GSDMD | gasdermin D | Pyroptosis |
| GSDME | gasdermin E | Pyroptosis |
| IL18 | interleukin 18 | Pyroptosis |
| IL1B | interleukin 1 beta | Pyroptosis |
| IL6 | interleukin 6 | Pyroptosis |
| NLRC4 | NLR family CARD domain containing 4 | Pyroptosis |
| NLRP1 | NLR family pyrin domain containing 1 | Pyroptosis |
| NLRP2 | NLR family pyrin domain containing 2 | Pyroptosis |
| NLRP3 | NLR family pyrin domain containing 3 | Pyroptosis |
| NLRP6 | NLR family pyrin domain containing 6 | Pyroptosis |
| NLRP7 | NLR family pyrin domain containing 7 | Pyroptosis |
| NOD1 | nucleotide binding oligomerization domain containing 1 | Pyroptosis |
| NOD2 | nucleotide binding oligomerization domain containing 2 | Pyroptosis |
| PJVK | pejvakin | Pyroptosis |
| PLCG1 | phospholipase C gamma 1 | Pyroptosis |
| PRKACA | protein kinase cAMP-activated catalytic subunit alpha | Pyroptosis |
| PYCARD | PYD and CARD domain containing | Pyroptosis |
| SCAF11 | SR-related CTD associated factor 11 | Pyroptosis |
| TIRAP | TIR domain containing adaptor protein | Pyroptosis |
| TNF | tumor necrosis factor | Pyroptosis |
| BAX | BCL2 associated X, apoptosis regulator | Necroptosis |
| BIRC2 | baculoviral IAP repeat containing 2 | Necroptosis |
| BIRC3 | baculoviral IAP repeat containing 3 | Necroptosis |
| CASP1 | caspase 1 | Necroptosis |
| CASP8 | caspase 8 | Necroptosis |
| CFLAR | CASP8 and FADD like apoptosis regulator | Necroptosis |
| FADD | Fas associated via death domain | Necroptosis |
| FAS | Fas cell surface death receptor | Necroptosis |
| FASLG | Fas ligand | Necroptosis |
| HMGB1 | high mobility group box 1 | Necroptosis |
| HSP90AA1 | heat shock protein 90 alpha family class A member 1 | Necroptosis |
| IL1A | interleukin 1 alpha | Necroptosis |
| IL1B | interleukin 1 beta | Necroptosis |
| MLKL | mixed lineage kinase domain like pseudokinase | Necroptosis |
| RIPK1 | receptor interacting serine/threonine kinase 1 | Necroptosis |
| RIPK3 | receptor interacting serine/threonine kinase 3 | Necroptosis |
| TNFRSF10A | TNF receptor superfamily member 10a | Necroptosis |
| TNFRSF10B | TNF receptor superfamily member 10b | Necroptosis |
| TNFSF10 | TNF superfamily member 10 | Necroptosis |
| TRADD | TNFRSF1A associated via death domain | Necroptosis |
| TRAF2 | TNF receptor associated factor 2 | Necroptosis |
| XIAP | X-linked inhibitor of apoptosis | Necroptosis |
| MAPK1 | mitogen-activated protein kinase 1 | Paraptosis |
| DAB2IP | DAB2 interacting protein | Paraptosis |
| MAPK14 | mitogen-activated protein kinase 14 | Paraptosis |
| PDCD6IP | programmed cell death 6 interacting protein | Paraptosis |
| ATF4 | activating transcription factor 4 | Paraptosis |
| GDF10 | growth differentiation factor 10 | Paraptosis |
| DDIT3 | DNA damage inducible transcript 3 | Paraptosis |
| MAPK8 | mitogen-activated protein kinase 8 | Paraptosis |
| MAP2K7 | mitogen-activated protein kinase kinase 7 | Paraptosis |
| EIF2AK3 | eukaryotic translation initiation factor 2 alpha kinase 3 | Paraptosis |
| XBP1 | X-box binding protein 1 | Paraptosis |
| ERN1 | endoplasmic reticulum to nucleus signaling 1 | Paraptosis |
| CDKN2A | cyclin dependent kinase inhibitor 2A | Curoptosis |
| CLS | cardiolipin synthase 1 | Curoptosis |
| DLAT | dihydrolipoamide S-acetyltransferase | Curoptosis |
| DLD | dihydrolipoamide dehydrogenase | Curoptosis |
| FDX1 | ferredoxin 1 | Curoptosis |
| LIAS | lipoic acid synthetase | Curoptosis |
| LIPT1 | lipoyltransferase 1 | Curoptosis |
| MTF1 | metal regulatory transcription factor 1 | Curoptosis |
| PDHA1 | pyruvate dehydrogenase E1 subunit alpha 1 | Curoptosis |
| PDHB | pyruvate dehydrogenase E1 subunit beta | Curoptosis |

**Supplementary Table 4 List of genes related to ferroptosis-related metabolic pathways**

| Gene Symbol | Description | Pathway |
| --- | --- | --- |
| ABCB10 | ATP binding cassette subfamily B member 10 | Iron metabolism |
| ABCB6 | ATP binding cassette subfamily B member 6 (Langereis blood group) | Iron metabolism |
| ABCB7 | ATP binding cassette subfamily B member 7 | Iron metabolism |
| ABCB8 | ATP binding cassette subfamily B member 8 | Iron metabolism |
| ABCG2 | ATP binding cassette subfamily G member 2 (Junior blood group) | Iron metabolism |
| ACO1 | aconitase 1 | Iron metabolism |
| ALAS1 | 5'-aminolevulinate synthase 1 | Iron metabolism |
| ALAS2 | 5'-aminolevulinate synthase 2 | Iron metabolism |
| ATP6AP1 | ATPase H+ transporting accessory protein 1 | Iron metabolism |
| BDH2 | 3-hydroxybutyrate dehydrogenase 2 | Iron metabolism |
| BMP6 | bone morphogenetic protein 6 | Iron metabolism |
| CD163 | CD163 molecule | Iron metabolism |
| CP | ceruloplasmin | Iron metabolism |
| CUBN | cubilin | Iron metabolism |
| CYBRD1 | cytochrome b reductase 1 | Iron metabolism |
| EIF2AK1 | eukaryotic translation initiation factor 2 alpha kinase 1 | Iron metabolism |
| ERFE | erythroferrone | Iron metabolism |
| FBXL5 | F-box and leucine rich repeat protein 5 | Iron metabolism |
| FECH | ferrochelatase | Iron metabolism |
| FLVCR1 | FLVCR heme transporter 1 | Iron metabolism |
| FLVCR2 | FLVCR heme transporter 2 | Iron metabolism |
| FTH1 | ferritin heavy chain 1 | Iron metabolism |
| FTHL17 | ferritin heavy chain like 17 | Iron metabolism |
| FTL | ferritin light chain | Iron metabolism |
| FTMT | ferritin mitochondrial | Iron metabolism |
| FXN | frataxin | Iron metabolism |
| GLRX3 | glutaredoxin 3 | Iron metabolism |
| HAMP | hepcidin antimicrobial peptide | Iron metabolism |
| HAVCR1 | hepatitis A virus cellular receptor 1 | Iron metabolism |
| HEPH | hephaestin | Iron metabolism |
| HFE | homeostatic iron regulator | Iron metabolism |
| HJV | hemojuvelin BMP co-receptor | Iron metabolism |
| HMOX1 | heme oxygenase 1 | Iron metabolism |
| HMOX2 | heme oxygenase 2 | Iron metabolism |
| HPX | hemopexin | Iron metabolism |
| IREB2 | iron responsive element binding protein 2 | Iron metabolism |
| ISCU | iron-sulfur cluster assembly enzyme | Iron metabolism |
| LCN2 | lipocalin 2 | Iron metabolism |
| LRP1 | LDL receptor related protein 1 | Iron metabolism |
| LTF | lactotransferrin | Iron metabolism |
| NCOA4 | nuclear receptor coactivator 4 | Iron metabolism |
| NDFIP1 | Nedd4 family interacting protein 1 | Iron metabolism |
| NUBP1 | NUBP iron-sulfur cluster assembly factor 1, cytosolic | Iron metabolism |
| PCBP1 | poly(rC) binding protein 1 | Iron metabolism |
| PCBP2 | poly(rC) binding protein 2 | Iron metabolism |
| SCARA5 | scavenger receptor class A member 5 | Iron metabolism |
| SFXN1 | sideroflexin 1 | Iron metabolism |
| SFXN2 | sideroflexin 2 | Iron metabolism |
| SFXN3 | sideroflexin 3 | Iron metabolism |
| SFXN4 | sideroflexin 4 | Iron metabolism |
| SFXN5 | sideroflexin 5 | Iron metabolism |
| SLC11A1 | solute carrier family 11 member 1 | Iron metabolism |
| SLC11A2 | solute carrier family 11 member 2 | Iron metabolism |
| SLC22A17 | solute carrier family 22 member 17 | Iron metabolism |
| SLC25A28 | solute carrier family 25 member 28 | Iron metabolism |
| SLC25A37 | solute carrier family 25 member 37 | Iron metabolism |
| SLC39A14 | solute carrier family 39 member 14 | Iron metabolism |
| SLC39A8 | solute carrier family 39 member 8 | Iron metabolism |
| SLC40A1 | solute carrier family 40 member 1 | Iron metabolism |
| SLC46A1 | solute carrier family 46 member 1 | Iron metabolism |
| SLC48A1 | solute carrier family 48 member 1 | Iron metabolism |
| STEAP1 | STEAP family member 1 | Iron metabolism |
| STEAP2 | STEAP2 metalloreductase | Iron metabolism |
| STEAP3 | STEAP3 metalloreductase | Iron metabolism |
| STEAP4 | STEAP4 metalloreductase | Iron metabolism |
| TF | transferrin | Iron metabolism |
| TFR2 | transferrin receptor 2 | Iron metabolism |
| TFRC | transferrin receptor | Iron metabolism |
| TMEM199 | transmembrane protein 199 | Iron metabolism |
| TMPRSS6 | transmembrane serine protease 6 | Iron metabolism |
| ACAA2 | acetyl-CoA acyltransferase 2 | Fatty Acid Biosynthesis |
| ACACA | acetyl-CoA carboxylase alpha | Fatty Acid Biosynthesis |
| ACACB | acetyl-CoA carboxylase beta | Fatty Acid Biosynthesis |
| ACLY | ATP citrate lyase | Fatty Acid Biosynthesis |
| ACSL1 | acyl-CoA synthetase long chain family member 1 | Fatty Acid Biosynthesis |
| ACSL3 | acyl-CoA synthetase long chain family member 3 | Fatty Acid Biosynthesis |
| ACSL4 | acyl-CoA synthetase long chain family member 4 | Fatty Acid Biosynthesis |
| ACSL5 | acyl-CoA synthetase long chain family member 5 | Fatty Acid Biosynthesis |
| ACSL6 | acyl-CoA synthetase long chain family member 6 | Fatty Acid Biosynthesis |
| ACSS2 | acyl-CoA synthetase short chain family member 2 | Fatty Acid Biosynthesis |
| DECR1 | 2,4-dienoyl-CoA reductase 1 | Fatty Acid Biosynthesis |
| ECH1 | enoyl-CoA hydratase 1 | Fatty Acid Biosynthesis |
| ECHDC1 | ethylmalonyl-CoA decarboxylase 1 | Fatty Acid Biosynthesis |
| ECHDC2 | enoyl-CoA hydratase domain containing 2 | Fatty Acid Biosynthesis |
| ECHDC3 | enoyl-CoA hydratase domain containing 3 | Fatty Acid Biosynthesis |
| ECHS1 | enoyl-CoA hydratase, short chain 1 | Fatty Acid Biosynthesis |
| FASN | fatty acid synthase | Fatty Acid Biosynthesis |
| HADH | hydroxyacyl-CoA dehydrogenase | Fatty Acid Biosynthesis |
| MECR | mitochondrial trans-2-enoyl-CoA reductase | Fatty Acid Biosynthesis |
| PC | pyruvate carboxylase | Fatty Acid Biosynthesis |
| PECR | peroxisomal trans-2-enoyl-CoA reductase | Fatty Acid Biosynthesis |
| SCD | stearoyl-CoA desaturase | Fatty Acid Biosynthesis |
| ATP12A | ATPase H+/K+ transporting non-gastric alpha2 subunit | Oxidative phosphorylation |
| ATP4A | ATPase H+/K+ transporting subunit alpha | Oxidative phosphorylation |
| ATP4B | ATPase H+/K+ transporting subunit beta | Oxidative phosphorylation |
| ATP6AP1 | ATPase H+ transporting accessory protein 1 | Oxidative phosphorylation |
| ATP6V0A1 | ATPase H+ transporting V0 subunit a1 | Oxidative phosphorylation |
| ATP6V0A2 | ATPase H+ transporting V0 subunit a2 | Oxidative phosphorylation |
| ATP6V0A4 | ATPase H+ transporting V0 subunit a4 | Oxidative phosphorylation |
| ATP6V0B | ATPase H+ transporting V0 subunit b | Oxidative phosphorylation |
| ATP6V0C | ATPase H+ transporting V0 subunit c | Oxidative phosphorylation |
| ATP6V0D1 | ATPase H+ transporting V0 subunit d1 | Oxidative phosphorylation |
| ATP6V0E1 | ATPase H+ transporting V0 subunit e1 | Oxidative phosphorylation |
| ATP6V0E2 | ATPase H+ transporting V0 subunit e2 | Oxidative phosphorylation |
| ATP6V1A | ATPase H+ transporting V1 subunit A | Oxidative phosphorylation |
| ATP6V1B1 | ATPase H+ transporting V1 subunit B1 | Oxidative phosphorylation |
| ATP6V1B2 | ATPase H+ transporting V1 subunit B2 | Oxidative phosphorylation |
| ATP6V1C1 | ATPase H+ transporting V1 subunit C1 | Oxidative phosphorylation |
| ATP6V1D | ATPase H+ transporting V1 subunit D | Oxidative phosphorylation |
| ATP6V1E1 | ATPase H+ transporting V1 subunit E1 | Oxidative phosphorylation |
| ATP6V1G1 | ATPase H+ transporting V1 subunit G1 | Oxidative phosphorylation |
| ATP6V1G2 | ATPase H+ transporting V1 subunit G2 | Oxidative phosphorylation |
| ATP6V1H | ATPase H+ transporting V1 subunit H | Oxidative phosphorylation |
| COX10 | cytochrome c oxidase assembly factor heme A: farnesyltransferase COX10 | Oxidative phosphorylation |
| COX11 | cytochrome c oxidase copper chaperone COX11 | Oxidative phosphorylation |
| COX15 | cytochrome c oxidase assembly homolog COX15 | Oxidative phosphorylation |
| COX4I1 | cytochrome c oxidase subunit 4I1 | Oxidative phosphorylation |
| COX5A | cytochrome c oxidase subunit 5A | Oxidative phosphorylation |
| COX5B | cytochrome c oxidase subunit 5B | Oxidative phosphorylation |
| COX6A1 | cytochrome c oxidase subunit 6A1 | Oxidative phosphorylation |
| COX6A2 | cytochrome c oxidase subunit 6A2 | Oxidative phosphorylation |
| COX6B1 | cytochrome c oxidase subunit 6B1 | Oxidative phosphorylation |
| COX6C | cytochrome c oxidase subunit 6C | Oxidative phosphorylation |
| COX7A1 | cytochrome c oxidase subunit 7A1 | Oxidative phosphorylation |
| COX7A2 | cytochrome c oxidase subunit 7A2 | Oxidative phosphorylation |
| COX7A2L | cytochrome c oxidase subunit 7A2 like | Oxidative phosphorylation |
| COX7B | cytochrome c oxidase subunit 7B | Oxidative phosphorylation |
| COX7C | cytochrome c oxidase subunit 7C | Oxidative phosphorylation |
| COX8A | cytochrome c oxidase subunit 8A | Oxidative phosphorylation |
| CYC1 | cytochrome c1 | Oxidative phosphorylation |
| LHPP | phospholysine phosphohistidine inorganic pyrophosphate phosphatase | Oxidative phosphorylation |
| NDUFA1 | NADH:ubiquinone oxidoreductase subunit A1 | Oxidative phosphorylation |
| NDUFA10 | NADH:ubiquinone oxidoreductase subunit A10 | Oxidative phosphorylation |
| NDUFA13 | NADH:ubiquinone oxidoreductase subunit A13 | Oxidative phosphorylation |
| NDUFA2 | NADH:ubiquinone oxidoreductase subunit A2 | Oxidative phosphorylation |
| NDUFA3 | NADH:ubiquinone oxidoreductase subunit A3 | Oxidative phosphorylation |
| NDUFA4 | NDUFA4 mitochondrial complex associated | Oxidative phosphorylation |
| NDUFA4L2 | NDUFA4 mitochondrial complex associated like 2 | Oxidative phosphorylation |
| NDUFA5 | NADH:ubiquinone oxidoreductase subunit A5 | Oxidative phosphorylation |
| NDUFA6 | NADH:ubiquinone oxidoreductase subunit A6 | Oxidative phosphorylation |
| NDUFA7 | NADH:ubiquinone oxidoreductase subunit A7 | Oxidative phosphorylation |
| NDUFA8 | NADH:ubiquinone oxidoreductase subunit A8 | Oxidative phosphorylation |
| NDUFA9 | NADH:ubiquinone oxidoreductase subunit A9 | Oxidative phosphorylation |
| NDUFAB1 | NADH:ubiquinone oxidoreductase subunit AB1 | Oxidative phosphorylation |
| NDUFB1 | NADH:ubiquinone oxidoreductase subunit B1 | Oxidative phosphorylation |
| NDUFB2 | NADH:ubiquinone oxidoreductase subunit B2 | Oxidative phosphorylation |
| NDUFB3 | NADH:ubiquinone oxidoreductase subunit B3 | Oxidative phosphorylation |
| NDUFB4 | NADH:ubiquinone oxidoreductase subunit B4 | Oxidative phosphorylation |
| NDUFB5 | NADH:ubiquinone oxidoreductase subunit B5 | Oxidative phosphorylation |
| NDUFB6 | NADH:ubiquinone oxidoreductase subunit B6 | Oxidative phosphorylation |
| NDUFB7 | NADH:ubiquinone oxidoreductase subunit B7 | Oxidative phosphorylation |
| NDUFB8 | NADH:ubiquinone oxidoreductase subunit B8 | Oxidative phosphorylation |
| NDUFC1 | NADH:ubiquinone oxidoreductase subunit C1 | Oxidative phosphorylation |
| NDUFS1 | NADH:ubiquinone oxidoreductase core subunit S1 | Oxidative phosphorylation |
| NDUFS2 | NADH:ubiquinone oxidoreductase core subunit S2 | Oxidative phosphorylation |
| NDUFS3 | NADH:ubiquinone oxidoreductase core subunit S3 | Oxidative phosphorylation |
| NDUFS4 | NADH:ubiquinone oxidoreductase subunit S4 | Oxidative phosphorylation |
| NDUFS6 | NADH:ubiquinone oxidoreductase subunit S6 | Oxidative phosphorylation |
| NDUFS7 | NADH:ubiquinone oxidoreductase core subunit S7 | Oxidative phosphorylation |
| NDUFV1 | NADH:ubiquinone oxidoreductase core subunit V1 | Oxidative phosphorylation |
| NDUFV2 | NADH:ubiquinone oxidoreductase core subunit V2 | Oxidative phosphorylation |
| PPA1 | inorganic pyrophosphatase 1 | Oxidative phosphorylation |
| PPA2 | inorganic pyrophosphatase 2 | Oxidative phosphorylation |
| SDHA | succinate dehydrogenase complex flavoprotein subunit A | Oxidative phosphorylation |
| SDHB | succinate dehydrogenase complex iron sulfur subunit B | Oxidative phosphorylation |
| SDHC | succinate dehydrogenase complex subunit C | Oxidative phosphorylation |
| SDHD | succinate dehydrogenase complex subunit D | Oxidative phosphorylation |
| TCIRG1 | T cell immune regulator 1, ATPase H+ transporting V0 subunit a3 | Oxidative phosphorylation |
| UQCR10 | ubiquinol-cytochrome c reductase, complex III subunit X | Oxidative phosphorylation |
| UQCR11 | ubiquinol-cytochrome c reductase, complex III subunit XI | Oxidative phosphorylation |
| UQCRB | ubiquinol-cytochrome c reductase binding protein | Oxidative phosphorylation |
| UQCRC1 | ubiquinol-cytochrome c reductase core protein 1 | Oxidative phosphorylation |
| UQCRC2 | ubiquinol-cytochrome c reductase core protein 2 | Oxidative phosphorylation |
| UQCRFS1 | ubiquinol-cytochrome c reductase, Rieske iron-sulfur polypeptide 1 | Oxidative phosphorylation |
| UQCRQ | ubiquinol-cytochrome c reductase complex III subunit VII | Oxidative phosphorylation |
| ALDOA | aldolase, fructose-bisphosphate A | Glycolysis |
| ALDOB | aldolase, fructose-bisphosphate B | Glycolysis |
| ALDOC | aldolase, fructose-bisphosphate C | Glycolysis |
| ENO1 | enolase 1 | Glycolysis |
| ENO2 | enolase 2 | Glycolysis |
| ENO3 | enolase 3 | Glycolysis |
| GAPDH | glyceraldehyde-3-phosphate dehydrogenase | Glycolysis |
| GCK | glucokinase | Glycolysis |
| GTPBP1 | GTP binding protein 1 | Glycolysis |
| HK1 | hexokinase 1 | Glycolysis |
| HK2 | hexokinase 2 | Glycolysis |
| HK3 | hexokinase 3 | Glycolysis |
| PFKFB1 | 6-phosphofructo-2-kinase/fructose-2,6-biphosphatase 1 | Glycolysis |
| PFKFB2 | 6-phosphofructo-2-kinase/fructose-2,6-biphosphatase 2 | Glycolysis |
| PFKFB3 | 6-phosphofructo-2-kinase/fructose-2,6-biphosphatase 3 | Glycolysis |
| PFKFB4 | 6-phosphofructo-2-kinase/fructose-2,6-biphosphatase 4 | Glycolysis |
| PFKP | phosphofructokinase, platelet | Glycolysis |
| PFKM | phosphofructokinase, muscle | Glycolysis |
| PGAM2 | phosphoglycerate mutase 2 | Glycolysis |
| PGK1 | phosphoglycerate kinase 1 | Glycolysis |
| PGK2 | phosphoglycerate kinase 2 | Glycolysis |
| PGM1 | phosphoglucomutase 1 | Glycolysis |
| PFM3 | parietal foramina 3 | Glycolysis |
| PKLR | pyruvate kinase L/R | Glycolysis |
| PKM | pyruvate kinase M1/2 | Glycolysis |
| SOD1 | superoxide dismutase 1 | Glycolysis |
| SOD2 | superoxide dismutase 2 | Glycolysis |
| SOD3 | superoxide dismutase 3 | Glycolysis |
| TPI1 | triosephosphate isomerase 1 | Glycolysis |
| ACLY | ATP citrate lyase | TCA cycle |
| ACO2 | aconitase 2 | TCA cycle |
| DLD | dihydrolipoamide dehydrogenase | TCA cycle |
| DLST | dihydrolipoamide S-succinyltransferase | TCA cycle |
| FH | fumarate hydratase | TCA cycle |
| IDH1 | isocitrate dehydrogenase (NADP (+)) 1 | TCA cycle |
| IDH2 | isocitrate dehydrogenase (NADP (+)) 2 | TCA cycle |
| MDH1 | malate dehydrogenase 1 | TCA cycle |
| MDH2 | malate dehydrogenase 2 | TCA cycle |
| OGDH | oxoglutarate dehydrogenase | TCA cycle |
| PDHA1 | pyruvate dehydrogenase E1 subunit alpha 1 | TCA cycle |
| PDHA2 | pyruvate dehydrogenase E1 subunit alpha 2 | TCA cycle |
| PDHB | pyruvate dehydrogenase E1 subunit beta | TCA cycle |
| PDK1 | pyruvate dehydrogenase kinase 1 | TCA cycle |
| PDK2 | pyruvate dehydrogenase kinase 2 | TCA cycle |
| PDK3 | pyruvate dehydrogenase kinase 3 | TCA cycle |
| PDPR | pyruvate dehydrogenase phosphatase regulatory subunit | TCA cycle |
| SDHAF1 | succinate dehydrogenase complex assembly factor 1 | TCA cycle |
| SDHB | succinate dehydrogenase complex iron sulfur subunit B | TCA cycle |
| SDHD | succinate dehydrogenase complex subunit D | TCA cycle |
| SUCLA2 | succinate-CoA ligase ADP-forming subunit beta | TCA cycle |
| SUCLG1 | succinate-CoA ligase GDP/ADP-forming subunit alpha | TCA cycle |
| SUCLG2 | succinate-CoA ligase GDP-forming subunit beta | TCA cycle |
| CHAC1 | ChaC glutathione specific gamma-glutamylcyclotransferase 1 | Glutathione synthesis |
| CHAC2 | ChaC glutathione specific gamma-glutamylcyclotransferase 2 | Glutathione synthesis |
| CNDP2 | carnosine dipeptidase 2 | Glutathione synthesis |
| GCLC | glutamate-cysteine ligase catalytic subunit | Glutathione synthesis |
| GCLM | glutamate-cysteine ligase modifier subunit | Glutathione synthesis |
| GGCT | gamma-glutamylcyclotransferase | Glutathione synthesis |
| GGT1 | gamma-glutamyltransferase 1 | Glutathione synthesis |
| GGT2 | gamma-glutamyltransferase 2, pseudogene | Glutathione synthesis |
| GGT3P | gamma-glutamyltransferase 3 pseudogene | Glutathione synthesis |
| GGT5 | gamma-glutamyltransferase 5 | Glutathione synthesis |
| GGT6 | gamma-glutamyltransferase 6 | Glutathione synthesis |
| GGT7 | gamma-glutamyltransferase 7 | Glutathione synthesis |
| GGTLC2 | gamma-glutamyltransferase light chain 2 | Glutathione synthesis |
| GSS | glutathione synthetase | Glutathione synthesis |
| LOC102724197 | inactive glutathione hydrolase 2 | Glutathione synthesis |
| OPLAH | 5-oxoprolinase, ATP-hydrolysing | Glutathione synthesis |
| ADSS1 | adenylosuccinate synthase 1 | Glutamine metabolism |
| ASNS | asparagine synthetase (glutamine-hydrolyzing) | Glutamine metabolism |
| ASNSD1 | asparagine synthetase domain containing 1 | Glutamine metabolism |
| CAD | carbamoyl-phosphate synthetase 2, aspartate transcarbamylase, and dihydroorotase | Glutamine metabolism |
| CPS1 | carbamoyl-phosphate synthase 1 | Glutamine metabolism |
| CTPS1 | CTP synthase 1 | Glutamine metabolism |
| CTPS2 | CTP synthase 2 | Glutamine metabolism |
| GFPT1 | glutamine--fructose-6-phosphate transaminase 1 | Glutamine metabolism |
| GFPT2 | glutamine-fructose-6-phosphate transaminase 2 | Glutamine metabolism |
| GLS | glutaminase | Glutamine metabolism |
| GLS2 | glutaminase 2 | Glutamine metabolism |
| GLUD1 | glutamate dehydrogenase 1 | Glutamine metabolism |
| GLUL | glutamate-ammonia ligase | Glutamine metabolism |
| GLYATL1 | glycine-N-acyltransferase like 1 | Glutamine metabolism |
| GLYATL1B | glycine-N-acyltransferase like 1B | Glutamine metabolism |
| GMPS | guanine monophosphate synthase | Glutamine metabolism |
| MECP2 | methyl-CpG binding protein 2 | Glutamine metabolism |
| NIT2 | nitrilase family member 2 | Glutamine metabolism |
| NR1H4 | nuclear receptor subfamily 1 group H member 4 | Glutamine metabolism |
| PFAS | phosphoribosylformylglycinamidine synthase | Glutamine metabolism |
| PHGDH | phosphoglycerate dehydrogenase | Glutamine metabolism |
| PPAT | phosphoribosyl pyrophosphate amidotransferase | Glutamine metabolism |
| SIRT4 | sirtuin 4 | Glutamine metabolism |
| DIO1 | iodothyronine deiodinase 1 | Selenium metabolism |
| DIO2 | iodothyronine deiodinase 2 | Selenium metabolism |
| DIO3 | iodothyronine deiodinase 3 | Selenium metabolism |
| GPX1 | glutathione peroxidase 1 | Selenium metabolism |
| GPX2 | glutathione peroxidase 2 | Selenium metabolism |
| GPX3 | glutathione peroxidase 3 | Selenium metabolism |
| GPX4 | glutathione peroxidase 4 | Selenium metabolism |
| MSRB1 | methionine sulfoxide reductase B1 | Selenium metabolism |
| SECISBP2 | SECIS binding protein 2 | Selenium metabolism |
| SELENOF | selenoprotein F | Selenium metabolism |
| SELENOI | selenoprotein I | Selenium metabolism |
| SELENOK | selenoprotein K | Selenium metabolism |
| SELENOM | selenoprotein M | Selenium metabolism |
| SELENON | selenoprotein N | Selenium metabolism |
| SELENOO | selenoprotein O | Selenium metabolism |
| SELENOP | selenoprotein P | Selenium metabolism |
| SELENOS | selenoprotein S | Selenium metabolism |
| SELENOT | selenoprotein T | Selenium metabolism |
| SELENOW | selenoprotein W | Selenium metabolism |
| SEPHS1 | selenophosphate synthetase 1 | Selenium metabolism |
| SEPHS2 | selenophosphate synthetase 2 | Selenium metabolism |
| SEPSECS | Sep (O-phosphoserine) tRNA:Sec (selenocysteine) tRNA synthase | Selenium metabolism |
| TXNRD1 | thioredoxin reductase 1 | Selenium metabolism |
| TXNRD2 | thioredoxin reductase 2 | Selenium metabolism |
| TXNRD3 | thioredoxin reductase 3 | Selenium metabolism |
